# Effects of high fat medium conditions on cellular gene expression profile: a network analysis approach 

**Published:** 2019

**Authors:** Hamid Asadzadeh-Aghdaei, Mohammad-Mehdi Zadeh-Esmaeel, Somayeh Esmaeili, Mostafa Rezaei Tavirani, Sina Rezaei Tavirani, Vahid Mansouri, Fatemeh Montazer

**Affiliations:** 1 *Gastroenterology and Liver Diseases Research Center, Research Institute for Gastroenterology and Liver Diseases, Shahid Beheshti University of Medical Sciences, Tehran, Iran*; 2 *Laser Application in Medical Sciences Research Center, Shahid Beheshti University of Medical Sciences, Tehran, Iran*; 3 *Traditional Medicine and Materia Medica Research Center, Department of Traditional Pharmacy, School of Traditional Medicine,, Shahid Beheshti University of Medical Sciences, Tehran, Iran*; 4 *Proteomics Research Center, Faculty of Paramedical Sciences, Shahid Beheshti University of Medical Sciences, Tehran, Iran*; 5 *Proteomics Research Center, Shahid Beheshti University of Medical Sciences, Tehran, Iran*; 6 *Firoozabadi Clinical Research Development Unit, Iran University of Medical Sciences, Tehran, Iran *

**Keywords:** HFM, Gene expression, PPI network

## Abstract

**Aim:**

This study aimed to evaluate high fat medium (HFM) effect on the gene expression profile of human Sk-hep1 cells and to determine critical differential proteins.

**Background:**

There is a correlation between high fat diet (HFD), obesity, and non-alcoholic fatty liver disease. Despite wide range of investigations, understanding molecular mechanism of HFD effect on onset and progression of NAFLD warrants further examination. In this study, network analysis is applied to obtain a clear perspective about HFD effects and NAFLD.

**Methods:**

Gene expression profiles of human Sk-hep1 cells treated with HFM versus controls were extracted from GEO. Data were analyzed by GEO2R where the significant and characterized DEGs were included in the PPI network. The top 10 nodes of query DEGs based on four centrality parameters were selected to determine central nodes. The common hub nodes with at least other one central group were identified as central nodes. Action map was provided for the introduced central nodes.

**Results:**

Heterogeneous nuclear ribonucleoprotein family including A1, A2/B1, D, R, and D-like, and five proteins (PRPF40A, SRSF1, PCF11, LSM8, and HSP90AA1) were introduced as differential proteins.

**Conclusion:**

mRNA processing and several biological terms including hypoxia and oxidative stress, apoptosis, regulation of cell morphology and cytoskeletal organization, and differentiation of micro tubes were introduced as dysregulated terms under HFM condition.

## Introduction

 Non-alcoholic steatohepatitis (NASH) first coined by Ludwig et al at 1980, is a serious liver disease caused by progression of non-alcoholic fatty liver disease (NFLD). NASH is associated with obesity and is characterized by liver inflammation, liver steatosis, and metabolomics syndrome (1-3). Several factors including fat accumulation, genetic differences, insulin resistance, and intestinal microbial flora are highlighted as effective factors that promote NAFLD-NASH process (4). A correlation has been reported and emphasized between intake of lipids and NASH (5). Two important hints about onset and progress of NASH are presented; the first is accumulation of fat in the liver, while the second hit is hepatic oxidative stress (6). There are different studies on the role of high fat diet and NASH onset and progress. The findings have been obtained from animal models. Xu et al. introduced a NASH animal model (Male Sprague–Dawley rats) through high fat dieting (7), while Romestaing et al. reported that long-term highly saturated fat diet did not induce NASH in Wistar rats (6). Carmiel-Haggai et al. observed that high fat diet leads to NASH-fibrosis progression in obese *fa/fa* Zucker rats. In this evaluation, the role of high fat diet is confirmed in progression of NAFLD (8). According to the review by Riordan and Nadeau,PI3K/AKT signalling, JAK/STAT signalling, PPAR signalling, and NF-κB signalling are important pathways whose dysregulation plays a significant role in progression of NAFLD (9).

Recently network analysis of diseases has attracted the attention of many scientists. In this approach, large numbers of genes or gene expression products which discriminate patients from controls interact to construct a network. In scale free networks, there are several elements that play function as crucial components in the interactome. These critical elements are useful tools to find the mechanism of diseases or those that may be responsible for onset and progression of diseases (10, 11). Previously, we reported a study on NAFLD rats which were treated with a high fatty diet. Gene expression changes of liver tissue were evaluated to find critically affected genes. The investigation was planned based on PPI network analysis which showed that GAPDH, PRDM10, TP53, AKT1, INS, ALB, SRC, MAPK1, ACLY, ACACA, DECR1, ACACB, MBOAT4, TNF, EHHADH and JUN genes are the important genes in progression of NAFLD through the high fat diet treatment (12). In the present study, gene expression profiles of human Sk-hep1 cells re treated with high fat diet are compared with controls to find the molecular mechanism of NASH induced by a high fat diet. 

## Methods

Six gene expression profiles of human Sk-hep1 cells including three samples treated with high-fat medium versus three samples as growth medium (GM)-treated cells were extracted from GEO. The samples are introduced as GSE109836 and GPL570 [HG-U133_Plus_2] Affymetrix Human Genome U133 Plus 2.0 Array. The incubation time was 12 hours and total RNAs from the two groups were extracted for the entire transcriptomic analysis. Data were analyzed by GEO2R and the top 250 DEGs were determined. Considering Fold change 2 (however threshold 1.5 also is enough) and P-value ≤ 0.05, the characterized DEGs were identified and included in the PPI network. Among the various isoforms, the DEGs with the maximum value of expression change were selected. The DEGs were included in the PPI network via STRING database by Cytoscape software. Due to poor interaction between the nodes of the constructed network, 100 neighbor genes from STRING database were added to the query DEGs to construct a scale free network. Top 10 nodes of query DEGs based on four centrality parameters; degree (D), betweenness centrality (BC), closeness centrality (CC), and stress (S) were selected to determine the central nodes. The common hub nodes with at least one other central group were identified as the central nodes. The connections between the central nodes were determined via a sub-network. The action map including binding, expression, activation, and inhibition was provided for the introduced central nodes. After evaluation, the potent central nodes were identified and introduced as possible biomarkers related to the effect of high fat nutrition on the treated cells. 

## Results

**Figure 1:**
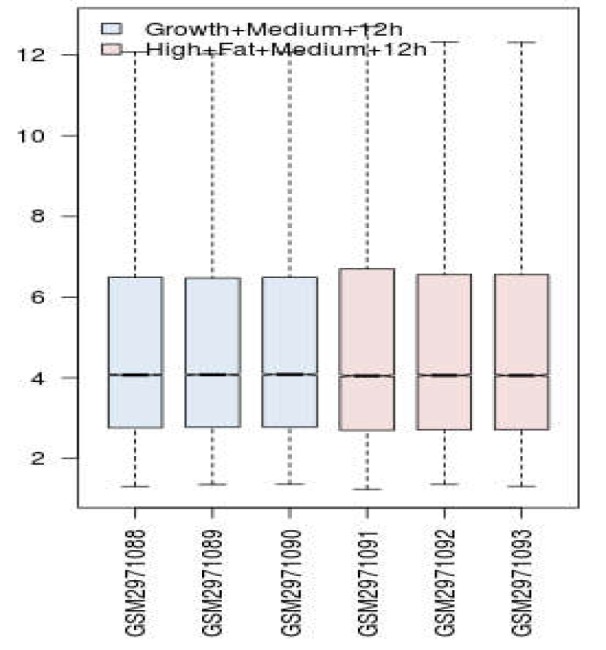
Distribution of gene expression values of samples represented by boxplot analysis; Blue and red refer to control and high fat treated groups. Profiles are median center and are matched statistically

**Figure 2:**
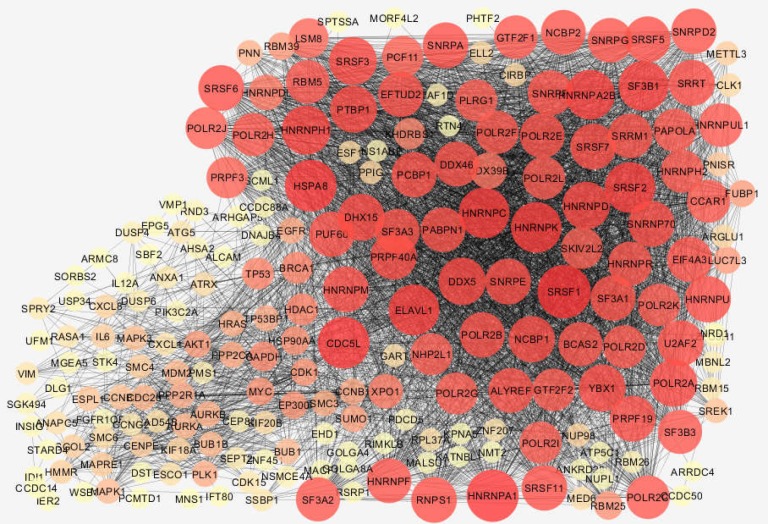
Main connected components of PPI network including 217 nodes (117 query DEGs and 100 neighbors) and 4597 edges; Nodes are layout based on degree value; larger size represent a high value of degree. Color change from yellow to red corresponds to increment in the degree value

Boxplot analysis is a suitable method to validate the possible comparability between samples. Since the distribution of data is median center (see figure 1), the gene expression profiles are comparable. 158 DEGs were imported in STRING database via Cytoscape software. Specifically, 134 out of 158 DEGs were recognized by STRING and included in the network. The constructed network was a poor network based on connections between nodes. Further, 34 isolated nodes and 10 nodes that were included in the four connected components were not included in the main connected component. Only 90 query DEGs were included in the main connected component. After adding 100 neighbors to the 134 DEGs, a network was constructed costing of 17 isolated DEGs and a main connected component. The main connected component contains 117 query DEGs and 100 added neighbor genes (see Figure 2). Ten central nodes of the network have been determined and presented in Table 1. Centrality parameters and description of the central nodes (extracted from STRING database) are reported in Table 1. 

**Table 1:** Top 10 hubs which are common with the 10 nodes based on closeness centrality; SRSF1, HNRNPA1, and HSP90AA1 are nodes that are common between top nodes based on four centrality parameters. HNRNPA2B1 is included in the hubs as well as the top nodes based on closeness centrality and stress

R	Gene	D	BC	CC	S	Description
1	SRSF1	101	0.021	0.607	15890	Pre-mRNA-splicing factor SF2, P33 subunit; Plays a role in preventing exon skipping, ensuring the accuracy of splicing, and regulating alternative splicing.
2	HNRNPA1	99	0.015	0.610	17274	Heterogeneous nuclear ribonucleoprotein A1; Involved in the packaging of pre-mRNA into hnRNP particles, transport of poly (A) mRNA from the nucleus to the cytoplasm.
3	HNRNPA2B1	94	0.008	0.590	10820	Heterogeneous nuclear ribonucleoproteins A2/B1; Heterogeneous nuclear ribonucleoprotein (hnRNP) that associates with nascent pre-mRNAs, packaging them into hnRNP particles.
4	HNRNPD	90	0.007	0.571	7986	Heterogeneous nuclear ribonucleoprotein D; Binds to RNA molecules that contain AU-rich elements (AREs) found within the 3'-UTR of many proto- oncogenes and cytokine mRNAs.
5	PRPF40A	88	0.002	0.551	4242	PRP40 pre-mRNA processing factor 40 homolog A; Plays a role in the regulation of cell morphology and cytoskeletal organization.
6	HNRNPR	84	0.006	0.555	4402	Heterogeneous nuclear ribonucleoprotein R; plays an important role in processing of precursor mRNA in the nucleus.
7	PCF11	79	0.001	0.522	1390	PCF11 cleavage and polyadenylation factor subunit; Component of pre-mRNA cleavage complex II.
8	LSM8	76	0.001	0.517	130	LSM8 homolog, U6 small nuclear RNA associated; Binds specifically to the U6 snRNA and is probably a component of the spliceosome.
9	HNRNPDL	68	0.002	0.522	3824	Heterogeneous nuclear ribonucleoprotein D-like; Acts as a transcriptional regulator. Promotes transcription repression. Promotes transcription activation in differentiated myotubes.
10	HSP90AA1	55	0.030	0.541	26472	Heat shock protein 90kDa alpha (cytosolic), class A member 1; Molecular chaperone that promotes the maturation, structural maintenance, and proper regulation of specific target proteins involved for instance in cell cycle control and signal transduction.

**Figure 3:**
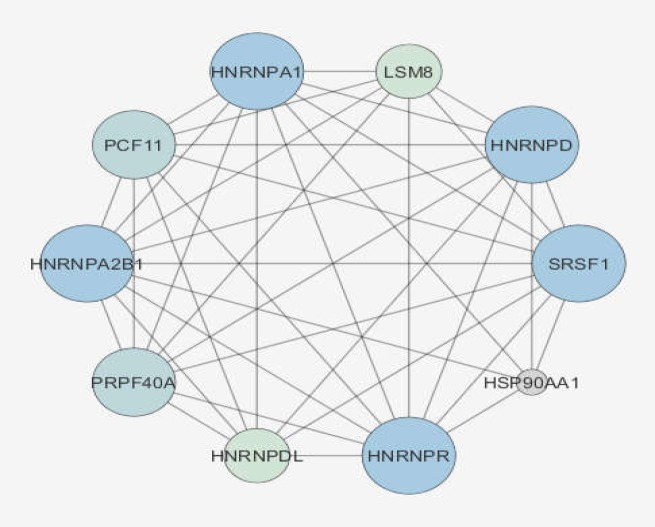
Connections between central nodes; the larger size refers to a higher value of degree. Light to dark color refers to increased betweenness

**Figure 4:**
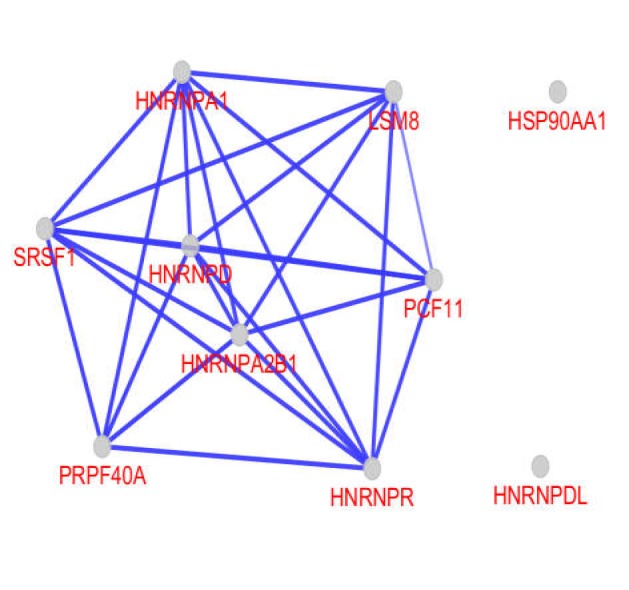
Action map including binding, expression, inhibition, and activation actions between central nodes; there was binding action only between genes, while HSP90AA1 and HNRNPDL were isolated

The connection between 10 central nodes is shown in the illustrated sub-network in Figure 3. Binding, expression, inhibition, and activation relationships between the central nodes are presented in Figure 4. As depicted in Figure 4, except for binding, there is no other regulatory relationships between the central nodes.

## Discussion

The gene expression profiles of high fat treated samples were comparable with controls based on the distribution of gene expression criterion with the differentially expressed genes included in a scale free network. A total of 10 central genes affected by high fat treatment were identified. Five genes among 10 central genes are different kinds of heterogeneous nuclear ribonucleoprotein family including A1, A2/B1, D, R, and D-like sub families. Heterogeneous nuclear ribonucleoprotein family are involved in management of resulted mRNAs from transcription. As with the introduced five heterogeneous nuclear ribonucleoproteins, the other genes are almost related to the processing of mRNAs. The fundamental cellular processes such as cell cycle are associated to the function of the introduced central nodes (see table 1). Except for HSP90AA1 and HNRNPDL, the other 8 central genes are connected to each other through binding type of action mode (see Figure 4). It seems that there are closed relationships between the central nodes to operate in response to intake of fat components. Apart from limited numbers, nucleocytoplasmic shuttling of the other heterogeneous nuclear ribonucleoproteins has been reported. One member of this family is HNRNPA1 which is a nuclear protein. This protein shuttles to cytoplasm and accrues with transcription inhibition (13). 

It is reported that PRPF40A expression as with COL1A1and UCP2 is involved in the biochemical pathways that are related to the hypoxia and oxidative stress. This finding is providing by Urszula Oleksiewicz et al based on an investigation on the non-small cell lung cancer (14). Up-regulation of PRPF40A in the pancreatic cancer is investigated and confirmed (15). As it is shown in the table 1, regulation of cell morphology and cytoskeletal organization is attributed to PRPF40A function.

SRSF1 is the other central gene which is presented in Table 1. This protein is characterized by a top degree value (110) and appears as a top hub-protein. Except HSP9oAA1 betweenness centrality of SRSF1 is the top value in Table 1. It is clear that this protein is the potent central protein in the network. SRSf1 plays important role in splicing (see Table 1). Over-expression of SRSF1 and its role in promotion of breast cancer has been reported by Olga Anczukow et al (2012). In this investigation, it is highlighted that SRSF1 in cooperation with MYC is involved in breast cancer progression (16). The proto-oncogenic property of SRSF1 and its up-regulation have been evaluated in several studies (17). Limin Zou et al. found that SRSF1 acts as an anti-apoptotic factor. Based on their report, this protein is related to leukemogenesis in pediatric ALL patients (18). 

PCF11 is the other protein introduced as a central protein. This protein is similar to many proteins that are involved in pre-mRNA 3′-end processing and transcription termination (19). 

LSM8 is the other critical protein that interacts with the rest of critical proteins except HNRNPDL (see table including LSM3-LSM8 which are involved in cis- and trans-splicing of mRNA (20).

HSP90AA1 gene encodes heat shock protein 90α (21) which has appeared as the last hub protein in Table 1. This protein is not included in the action map and has remained as an isolated protein (see Figure 4). A significant reduction in HSP90AA1 mRNA level in human liver is reported for alcoholic fatty liver disease (AFLD) patients in comparison with normal samples (22). 

The literature findings suggest that the central nodes play a critical role in the promotion of high fat medium effects in the body. It seems that further investigation can be useful to introduce valuable biomarkers for diagnosis of disease and follow up of patients with fatty liver disorder. The analysis revealed that the heterogeneous nuclear ribonucleoprotein family and mRNA processing are the crucial proteins and biochemical pathways which are involved in the onset and progression of fatty liver disease. Several biological terms including hypoxia and oxidative stress, apoptosis, regulation of cell morphology and cytoskeletal organization, and differentiation of micro tubes were highlighted as processes dysregulated under high fat diet condition. The findings can suggest the discovery of a possible biomarker associated with the fatty liver disorder.
